# Autobiographical emotional induction in older people through popular songs: Effect of reminiscence bump and enculturation

**DOI:** 10.1371/journal.pone.0238434

**Published:** 2020-09-02

**Authors:** Marco A. López-Cano, Beatriz Navarro, Marta Nieto, Fernando Andrés-Pretel, José M. Latorre

**Affiliations:** 1 Department of Psychology, University of Castilla La Mancha, Albacete, Spain; 2 Unidad de Investigación, Gerencia de Atención Integrada de Albacete, Fundación del Hospital Nacional de Parapléjicos, Albacete, Spain; Murcia University, Spain, SPAIN

## Abstract

**Aims:**

This study is based on two experiments, the first, with an exploratory character. The aim of which is to assess the capacity of native vs international pop songs (NAT vs INT) from two consecutive life stages, Reminiscence bump (RB) and the immediately subsequent period (No reminiscence bump, NORB) to elicit positive emotions and autobiographical memories.

**Method:**

A total of 15 middle-aged adults and 15 older adults participated in Experiment 1 (E1). Emotionality, song familiarity and associated autobiographical memories were assessed. Each participant was exposed to 20 randomly selected age-specific songs. Pre-and post-test measures of mood state were also included. Experiment 2 (E2) focused on late adulthood, using a sample of 35 persons. The experimental design was similar to that used in E1. However, this second experiment also included an analysis of the types of autobiographical memories generated by the experimental task and a study of their relationship with the characteristics of the songs, their familiarity and the emotions they produced, and the number of trials. The aim was to delve into the effects that influence the effectiveness of the induction procedure, particularly as regards emotional positivity and memory specificity.

**Results:**

Regarding age effect, E1 results varied: under some conditions, emotionality showed no difference between groups, others showed positive older adult bias. In E2, the analysis of the relationships between memory types and the selected variables suggests the latter are not useful predictors of differences between memory types. The study design yielded a relatively high level of memory specificity and emotional positivity.

**Conclusion:**

The findings question positivity bias in the elderly. RB music produces different effects depending on age. Enculturation can be an important mediating factor in emotionality and memory. Finally, experimental design improves specific memory and positivity.

## Introduction

This study investigates how popular songs can induce autobiographical emotional memories. The findings provide data that allow us to broaden the perspective and to more accurately use the variables involved in mood induction procedures based on music and memory, especially with regard to the effect of age, reminiscence bump, enculturation and emotional positivity, opening new avenues for future research. Furthermore, the positive results obtained related to specific memory and mood are promising in relation to the field of health care and well-being of older adults.

Music plays a key role in most people’s lives, frequently being used to explore and regulate emotions [1,2]. A number of studies have suggested that the emotional responses evoked by music may be related to autobiographical memories [3–5]. In this line, it has been reported that some responses to music are influenced by learning [6], and also appear to represent a mixture of cognitive and emotional components, suggesting a type of conditioning that is not always consciously available, although these responses evoke emotional memories related to specific places, events or experiences [7]. It is also worth noting that music is always heard in a social context, with or without the presence of other individuals, and with other activities taking place that have their own complex sources of meaning and emotion. In other words, the emotional response to music is coloured, and sometimes determined, by these contextual factors [8].

### Autobiographical memory

Autobiographical memory (AM) refers to a knowledge base of personal information that includes specific, episodic memories of past events and more conceptual, self-related information [9,10]. Autobiographical knowledge is hierarchically organized across different levels of specificity. At the general level, we can find extended memories, which are general memories associated with events that last more than a day (e.g., “the weekend I spent in London”), and categorical memories, which are general, repeated events grouped together in a category (e.g., “Christmas dinners with my family”). At the lowest level are specific AMs, which are personally significant memories associated with a time and place that lasted a day or less than a day (e.g., “when I get married”).

Apart from this type of hierarchical organization, the AM content depends on the age of encoding of memories. The distribution of autobiographical memory over the lifespan can be observed in many situations, but is more evident when individuals freely remember events from their own lives and then date them [11]. These distributions typically have three main components. The first of these is called *infantile amnesia* and refers to the lack of memories from the first five years of life. The second component is the *reminiscence bump* (RB), referring to how people remember relatively large numbers of events from their adolescence and early adulthood (between the ages of around 15 and 30) [12]. Importantly, due to the large number of significant events that tend to take place during this life stage (e.g., graduation, first romantic relationships, childbirth), individuals’ most positive memories typically refer to this period [13,14]. Nonetheless, recent research suggests that the “bump” has been misunderstood, as it is often considered a unitary phenomenon, when actually the size and the temporal location of the bump are sensitive to the cues and assessment methods used to elicit memories [15]. The RB is generally followed by a period with fewer memories. Finally, the third component is what is known as the recency effect [9], which refers to the most accurately recalled events being those from recent years.

The capacity to retrieve specific autobiographical memories is an indicator of emotional well-being [10]. In contrast, difficulty in retrieving specific AMs (i.e., overgeneral memory) has been associated with certain types of psychopathology, such as depressive disorders or post-traumatic stress disorder [10,16]. Hence, autobiographical memories can be considered to play a core role in emotion regulation, [17–19]. Prior findings have shown that retrieval of positive specific events is a suitable procedure to reduce negative mood states and improve depressive symptoms [20,21].

### Musical cues in emotion induction and autobiographical memories

In the context of the present research, there are numerous factors that affect the subjective responses related to music, emotion and memory [4]. Our study thus focuses on some of the variables that have been considered most significant in the current literature [21–24], such as memory, familiarity, valence, arousal and specificity

Autobiographical recall has been shown to be one of the most effective ways of inducing affective states [5], and involves generating personal emotional memories to reactivate emotions from the original emotional experience [26]. This means that by focusing a person’s attention on sensory events and responses (sights, smells, sounds, physiological and behavioral reactions) that occurred during an emotional experience, we can maximize the likelihood of reliving an intense emotional experience [27]. The vividness of these memories at the level of encoding and retrieval involves numerous elements, such as the clarity, details and strength of the memory, as well as the arousal, valence, significance and personal involvement in an event. Personality traits also appear to increase the likelihood of a vivid memory being consolidated and stabilized over time [3].

The evidence suggests that emotional music clips can act as a highly successful tool for generating autobiographical memories [22,25,28,29]. This mnemonic enhancement may be associated with the subjective familiarity of musical cues, where highly familiar stimuli have been related to richer memory representations [22]. In this line, neuroimaging research has found that familiarity seems to be a key factor in getting listeners emotionally engaged with the music [30].

Music is a useful tool in this research area given its potential to evoke strong emotions and has yielded results that are compatible with the RB literature using musical stimuli. For example, Schulkind et al. [28], cited by Shubert [31], reported a RB with an age range from 14 to 28 years, using excerpts of popular songs as memory cues in groups of younger and older adults. As in much of the RB research, studies on autobiographical musical memories typically use stimuli associated with a particular calendar year. The selection of the excerpts is thus biased towards certain forms of popular music because the time such music is popular tends to be transitory. Annual music sales charts provide a relatively simple way to select cues associated with a small time range (generally a question of months), and so a reasonable degree of experimental control can be achieved by using popular music excerpts as a stimulus specific prompt [31].

A number of studies have reported that using music to elicit emotions is one of the most effective methods of emotion induction. Nonetheless, one of the principal limitations of these procedures is the lack of standardization, given the difficulty of controlling for the large quantity of individual variables that have an impact on the results, such as the subjectivity music invokes in emotions and autobiographical memories. This has led to a diversity of methodologies, complicating the interpretation of the data obtained and impairing the effectiveness of the procedures implemented [4,32,33]. Considerable variations across the studies reviewed have been detected. For example, participants’ musical training or mood state is not always controlled for. As regards procedural differences, the degree of popularity of the musical stimuli tends to vary, with some studies using unfamiliar music and others well-known hits, which may impact the induction process given that familiarity is considered a key factor in making listeners emotionally engaged with music [30]. The experimental tasks are conducted in both individual and group formats, with a contagion effect in the latter potentially affecting the emotional responses [4]. Finally, the effect of fatigue related to the number of stimuli used is seldom controlled for, which would seem to be significant given its influence on cognitive performance and brain activity associated with the task in question [34,35].

Focusing on the effectiveness of mood induction procedures based on music and memory (MIPMMs), the limitations mentioned above and the lack of precision in the consideration of the factors that influence the relationships between music, memory and emotion might also explain the disparity in the findings on autobiographical memories, with percentages of memory retrieval ranging from 30 to 78 [22–25,28].

### The current study

As an initial approach to a deeper analysis of the characteristics influencing the effectiveness of MIPMMs, the current study considered two variables that have received scant attention in this field. First is the age of participants, with regard to adulthood and the life period to which the musical clues belong, since most previous works either fail to define late adulthood or do not include middle-aged adults in the study [e.g., 25,26,28–30]. Most studies in this field focus on comparing responses between young and older adults, with scant attention being paid to middle-aged persons. Ford et al. [22] found significant age-related differences in the effects of song familiarity, where familiarity was associated with enhanced memory detail in young adults and affective positivity in older adults. Regarding emotionality (valence), the literature has traditionally reported a positive bias related to an increase in age [36]. It is also important to note that the aging process affects normal brain activity and generally changes brain response to stimuli [37].

Second is the importance of the variable of musical enculturation in the positive autobiographical memories evoked by the music. The largely unconscious process of acquiring culturally rooted understandings has been labeled enculturation [38]. Enculturation is defined as the process by which a person acquires the understandings and beliefs of a particular society from infancy without any special training. Enculturation can influence our understanding of the world in a variety of ways related to our membership in a given society and our sense of identity.

Cognitive psychologists typically treat memories and recall as processes that take place wholly within the individual, without considering that an individual’s memory is embedded in a broader social, cultural and historical setting [39]. Authors such as Bluck [40] suggest that culture is an influencing factor and is present in both a person’s internal and external environment. Collective memories can involve small groups, such as families, or large groups, such as nations [41].

In this regard, it has been suggested that exposure to culturally different music increases preference ratings [42]. Previous studies have indicated that cortical response patterns in the encoding and retrieval of familiar and unfamiliar music are different, and music that is more culturally familiar is recognized more easily [43,44]. To our knowledge, studies on MIPMMs have paid little attention to the concept of enculturation, despite its implications, especially with regard to intercultural generalization. Moreover, deeper knowledge of this particular element could be key in the successful application of these procedures in the field of healthcare as a novel method to generate positive effects on older adults’ well-being and quality of life. Accordingly, the two studies that comprise the current work are focused on assessing the capacity for mood induction and autobiographical memory retrieval of songs from “different cultures” and from two different life periods (RB/NORB) in middle-aged and older adults. With this general aim in mind, the following hypotheses were proposed:

#### First hypothesis (E1 and E2)

In line with previous studies, we expected to find higher levels of familiarity, emotionality and autobiographical memory in E1 and memory specificity in E2 associated with stimuli from the RB period (see findings comparing younger and older adults in Ford et al. [29]) and with NAT. Regarding the memory variable (E1), it should be noted that our main interest was its covert influence on the emotions induced by the music.

#### Second hypothesis

The aim of E1 was to study, in adulthood, the effect of age on the emotional responses generated by the music, and on autobiographical memory and familiarity, depending on the life period and origin (NAT/INT) of the stimuli. To the best of our knowledge, in the field of music and autobiographical memory, little data has been published, especially with reference to comparisons between middle-aged and older adults. Janssen et al. [45], with a sample aged between 10 and 70 years, and using words as memory retrieval cues, found strong evidence for a RB, with peaks between the ages of 13 and 18. Their findings also revealed significant differences, with greater encoding intensities between the age groups of 61 to 70 and 41 to 50 years. Hence, higher levels of memories associated with the RB were expected to be found among the older adults in our study. As regards emotionality (valence), although it has traditionally been supposed that the older we are, the more positively biased are our memories [36], a recent work using film clips with differing valences reported results that cast doubt on this assumption [46], opening up fresh debate on emotional processing in adulthood. In this line, we predicted that our findings would vary depending on the stimuli and methodology used in the experiments. As regards familiarity, to the best of our knowledge, only one study using a similar methodology has addressed the effect of age on this variable across the different stages of adulthood [47], finding higher memory levels in middle-aged adults compared to older adults.

#### Third hypothesis

With the idea of delving deeper into the effectiveness of MIPMMs, especially with regard to the positivity and memory specificity induced by the songs, the aim of E2 was to study the relationship between the features of the musical cues, emotions and type of autobiographical memory. There is a broad consensus on the existence of a positive bias among middle-aged adults [29] in the context of music, even when this is negatively valenced [48]. Nonetheless, academic research is still to determine why positive memories are more easily retrieved than negative ones [29]. In our study, we expected to find a positive relationship between the subjective assessments of the emotions induced by the pop music and the valence of the autobiographical memory.

## Method, Experiment 1

### Participants

Fifteen healthy middle-aged adults were recruited, *M* age = 46.60, *SD* = 7.78, range = 35–59 years; 73% female, and 15 healthy older adults, *M* age = 71.33, *SD* = 5.23, range = 63–80; 66% female. General state of health, auditory functions and years of academic education are shown in Table 1 (E1 and E2).

**Table 1:** General state of health, auditory functions and years of academic education, studies 1 and 2.

	Experiment 1		Experiment 2
	Middle-aged adults	Older adults	
General state of health			
Low	-	20.00	-
Good	80.00	60.00	82.90
Very good	20.00	20.00	17.10
Auditory functions			
Low	20.00	20.00	17.10
Good	46.70	46.70	62.90
Very good	33.30	33.30	20.00
Education (mean±SD[range])	15.40±2.90 (8–17)	8.33±2.97 (5–12)	10.48±4.68 (5–17)

Note 1 = Values are expressed as percentages, except for mean±SD (min.-max.). Following Platz et al. [24], current state of health and auditory functions were self-reported on a 4-point Likert-type scale (0 = very poor, 3 = very good). The mean value for education refers to the years of academic education.

Exclusion criteria for all participants were: (a) following El Haj et al. [49], more than four years of musical practice (equivalent to 1st grade of music school); (b) being under the age of 35 years, since the study design did not allow the use of more than 10 non-RB stimuli and an individual aged 34 years would need to be exposed to stimuli from 2017 (year the study was conducted); (c) being ill and /or having auditory disorders that would make it difficult to undertake the tasks; and (d) no active signs of cognitive deficit at the time of assessment. The fourth exclusion criterion was measured using the Spanish version of the Mini-Mental Status Examination (MMSE) [50]. Apart from the participants selected for the study (N = 30), four participants were excluded under these criteria: two because they had completed musical studies and two due to problems with the recording equipment during the experimental task. In both studies, all participants gave written informed consent in accordance with the requirements of the Helsinki Declaration, current Spanish legislation (Law 14/2007 on biomedical research) following the recommendations of Agreement 06/2016 of the Clinical Research Ethics Committee (CREC). The protocol was approved by the Clinical Research Ethics Committee of the Castilla-La Mancha Health Service. Spanish was the first language of all the participants.

### Materials

#### Mood state

The Positive (PA) and Negative Affect (NA) Schedule [51] was used to assess pre- and post-test mood state. The PANAS has 10 adjectives measuring PA, and 10 adjectives measuring NA. The PANAS was designed to measure affect in various contexts such as at the present moment, the past day, week, or year, or in general (on average). Thus, the scale can be used to measure state affect, dispositional or trait affect, emotional fluctuations throughout a specific period of time, or emotional responses to events [52]. The scale comprises 20 Likert-type items (1 = very slightly or not at all, 5 = extremely). Our study used the Spanish adaptation by PANAS [53], the psychometric properties of which were validated with a Cronbach’s alpha of 0.92 for the positive affect subscale and 0.88 for the negative affect subscale.

#### Main study variables

*Musical stimuli*. The pop songs used were a compilation of chart hits, one from each year between 1952 and 2017. A total of 130 excerpts were selected (65 excerpts of native popular music, with the exception of 1962, for when the song is by a Cuban group, but sung in Spanish and 65 excerpts of international pop). Each was 30 seconds in length, as in similar studies [22,25]. The songs were selected using various music databases [54–58]. As a selection criterion, in order to enhance song familiarity, all the songs had reached number one on the pop charts consulted on the year of their release. The international songs from the years 1952–1959 were selected from the database “Wikipedia: Lists of Billboard Hot 100 number-one songs (USA, 2017)”, as the Spanish charts for those years barely contained international pop music. To avoid confusions, we attempted not to repeat groups or songwriters, although for some years this was not possible due to the lack of variety in the songwriters of number-one hits in certain years (1953 and 1955; 1978 and 1980; 1964 and 1965; 1976 and 2000). In addition, we did not select songs that were at the top of the charts in consecutive years. Considering these criteria, months were randomly selected until an appropriate stimulus was found.

The excerpts selected were easily recognizable parts, such as the chorus. Each participant listened to 10 song excerpts corresponding to the first five years of their RB and 10 from the five years immediately after the end of their RB, in such a way that, for example, an individual born in 1957, with a current age of 59 or 60, was exposed to a selection from the years 1972–1976 (RB) and 1988–1992 (NORB). This range was determined in accordance with the approximate standard RB period of ages 15–30 [59].

*Valence and arousal*. Following previous studies [24,28], valence and arousal were measured using the Self-Assessment Manikin [60]. This is a self-reported questionnaire that evaluates emotional responses, measuring affective valence, arousal and dominance or emotional control. Considering the dimensional structure of affect [61], we administered the items measuring valence and arousal. These two dimensions are those most commonly used in the literature [51,62]. Thus, participants rated, on a 9-point Likert-scale, how unpleasant (1) or pleasant (9) and how relaxed (1) or aroused (9) they felt while listening the songs.

*Familiarity and autobiographical memories*. Familiarity with the music was rated on a 3-level Likert-type (1 = unfamiliar, 3 = very familiar), as used by Vuoskoski et al. [5].

To assess the autobiographical memories associated with the musical stimuli, a dichotomous question was asked: memory evoked by the music, yes/no. Following the model proposed by Conway et al. [9], the participants were explained the difference between general and specific memories, where “general events” refers to events lasting days, weeks or months and which represent knowledge on personal goals, achievements and activities. These general events may also refer to actions repeated over time. “Specific events” refers to periods of time lasting seconds, minutes or hours. They tend to be associated with sensory details, vivid images and emotions. It is worth noting that the memories was not recorded. Given the exploratory nature of the study, the main aim of the experimental design was to associate the memory with the music in order to produce a positive effect on mood state and memory activation.

### Procedure

The study was announced on social media and at community centers in the city of Albacete (Spain). The management teams at these centers collaborated in the study. Permission for the study was requested from both the centers and the University of Castilla-La Mancha (UCLM). The tests were administered between March and May 2017 by the main author of the present article.

#### Phase 1

To avoid possible effects of fatigue and boredom, the study was divided into two phases. The participants completed the two phases at intervals ranging from one to seven days. In line with similar studies [5], before the experimental session, in order to minimize the effect of demand characteristics, the participants were told that the aim of the research was to study the relationship between music and cognitive processes, perception and memory. They were not informed about the role of emotion in the experimental tasks. We then collected sociodemographic data, self-reported state of health and auditory functions and self-rated musical experience. We also administered the MMSE to the group of older adults to rule out any cognitive impairment.

#### Phase 2

This phase was conducted individually in a tranquil environment in order to create an atmosphere of intimacy and avoid distractions. Before the task was conducted, we administered tests to measure mood state. Using the PANAS scale, the participants were requested to assess how they had felt over the last week, including at the current time. A trial run of the experimental task was conducted following the procedure described below:

*Evaluation of the emotions associated with the music*. Participants were given a detailed description of each of the two dimensions on the SAM scale (valence and arousal), emphasizing that they were being asked to assess the emotion evoked by listening to the music.

*Autobiographical memory*. After replying to the question about their familiarity with the music, participants were asked whether the song had generated any autobiographical memory. Participants were then given an explanation the differences between the memory categories previously described and were told that the events recalled should be ones they had personally experienced and that were related to a specific event.

At the beginning of the task, the participants were instructed to attempt to relax and concentrate on their breathing for about a minute. After the test was finished, they were administered the PANAS questionnaire to assess the general effect of the task on their mood state and were asked to evaluate how they felt at that moment.

The experimental task design was implemented using E-prime version 2.0, following the flow diagram shown in S1 Fig. Phase A (time limit) was not included in this study, nor were I, J and K (part of E2), as the responses to the memory were not recorded. The trials consisted of exposure to 20 randomly selected pop songs and the answering of various questions previously described. Before the test and in order to avoid any possible sensory interference, the participants were asked to close their eyes while each excerpt was played, given that emotional information from a concurrent but non-attended sensory channel influences the processing of the attended modality [63]. Such interferences were not usually taken into account in previous research. Participants carried out the task and listened to the songs on a laptop, using earphones on which they could adjust the volume.

### Data analysis

The statistical analyses were conducted using SPSS v.23 and the graphics were generated using Excel 16.0. Lucidchart was used to design the diagram of the test sequence. Alpha was set at .05. For the descriptive analyses of the responses regarding the autobiographical memory, the variable was organized into two categories, “memory” and “no memory”. Finally, the participants were divided into two age groups: middle-aged adults (35 to 59 years) and older adults (60 to 80 years). The effect size was calculated using Cohen’s d in all the t-tests. The other analyses are described in the corresponding sections.

## Results

### Effect of age, life stage and origin of music

To evaluate the effect of age, life stage (LS) and origin of the music (OM) on valence, arousal, familiarity and memory, a 2x2x2 mixed factorial design was used, where age group (middle aged adults–older adults) acted as a between-subjects variable, and LS (RB-NORB) and OM (NAT-INT) were within-subjects variables. The Bonferroni correction was used in the student’s t-tests. Table 2 shows the results (mean scores).

**Table 2:** Mean scores for the variables of memory, familiarity, valence, arousal in relation to life stage (reminiscence bump-no reminiscence bump) and origin of the music (native music–international music).

	Middle-aged adults				Older adults			
	Reminiscence bump	No reminiscence bump	Native songs	International songs	Reminiscence bump	No reminiscence bump	Native songs	International songs
	*M(SD)*	*M(SD)*	*M(SD)*	*M(SD)*	*M(SD)*	*M(SD)*	*M(SD)*	*M(SD)*
Memory	7.13(2.20)	3.80(2.14)	5.67(2.16)	5.27(1.71)	8.07(2.25)	4.73(2.60)	7.87(2.17)	4.93(2.37)
Familiarity	2.79(.21)	2.47(.45)	2.71(.23)	2.56(.38)	2.52(.24)	1.95(.30)	2.52(.25)	1.95(.25)
Valence	6.71(1.40)	6.53(1.33)	6.58(1.45)	6.66(1.30)	7.45(.70)	6.23(.68)	7.53(.84)	6.15(.90)
Arousal	5.87(1.19)	5.50(1.05)	5.83(1.12)	5.54(1.11)	6.49(1.26)	5.23(1.10)	6.07(1.69)	5.65(.81)

The mean values are expressed as *M*(*SD*). The scale for the memory was the total number of songs that evoked memories by condition, taking into account these are within-subject comparisons and the memories described refer to sets of 10. Thus, on the one hand, they are compared according to whether they belong to the reminiscence bump and, on the other, depending on whether they are native or international. For example, the reminiscence bump condition comprised 5 native stimuli and 5 international stimuli. For familiarity, we used a 3-point Likert scale and for valence and arousal, a 9-point Likert scale.

At between-subjects level, a significant main effect for age group was found only on familiarity, with the middle-aged adults scoring higher on response on all variables. At within-subjects level, our results revealed a significant main effect of LS on all the dependent variable scores with higher response levels in the RB condition. As regards OM, a significant effect was found on all the variables (with higher scores on the NAT condition), except for arousal (see results in Table 3). Regarding the LS×age group effect, the results revealed significant interactions for the SAM variables, where the LS factor generated higher levels of valence in the RB period compared to NORB, with significant differences and a higher effect between both periods in the older adult group, *t*(14) = 6.56, *p* < .001, *d* = 1.74, while the differences in the middle-aged adult group were non-significant, *t*(14) = 0.77, *p* = .454. The comparisons of responses between groups in the same level were non-significant, evaluations in the RB stage were higher in the older adults *t*(28) = -1.82, *p* = .078, and in the NORB stage, the middle-aged adults exhibited more positive evaluations *t*(28) = 0.78, *p* = .443. On arousal, the RB period generated higher response levels than NORB, with the effect being higher in the older adults *t*(14) = 6.96, *p* < .001, *d* = 1.07, compared to their middle-aged counterparts, where the comparisons were non-significant, *t*(14) = 1.74, *p* = .104. There was no interaction in the memory variable, with the older adults showing higher response rates on both levels. The between-group comparisons within each LS level revealed no significant differences: RB memory, *t*(28) = -1.15, *p* = .260, NORB memory, *t*(28) = -1.07, *p* = .293 . With regard to familiarity, LS generated a decline in response levels from RB to NORB, with the effect being much higher in the older adults, *t*(14) = 6.51, *p* < .001, *d* = 2.10 compared to the middle-aged adults, *t*(14) = 3.33, *p* = .005, *d* = .91. The between-groups comparisons within each LS level revealed significant differences in each life period, with a slightly higher effect in the RB period, *t*(28) = 3.29, *p* = .003, *d* = 1.36, NORB period, *t*(28) = 3.79, *p* = .001, *d* = 1.20.

**Table 3:** Repeated measures analysis.

			Age		Life stage		Origin of the music		Origin of the music*Life stage		Age *Life stage		Age *Origin of the music	
	Middle-aged adults *M(SD)*	Older adults *M(SD)*	*F*	ηp²	*F*	ηp²	*F*	ηp²	*F*	ηp²	*F*	ηp²	*F*	ηp²
Memory	10.93(3.59)	12.80(4.16)	1.73	.06	54.05***	.66	29.71***	.51	1.78	.05	0.00	.00	17.16***	.38
Familiarity	2.63(.30)	2.23(.21)	18.10***	.39	46.90***	.63	60.93***	.68	3.86	.12	3.77	.06	21.40***	.43
Valence	6.62(1.28)	6.84(.59)	0.36	.01	21.29***	.43	9.47**	.25	0.55	.02	11.53**	.29	11.96**	.30
Arousal	5.68(1.04)	5.86(1.13)	0.20	.01	34.36***	.55	3.01	.10	1.87	.06	10.43**	.27	.12	.004

***p < .001,

**p < .01,

*p < .05. The mean values are expressed as *M*(*SD*). The life stage or period and origin of the music comprise the levels of reminiscence bump—no reminiscence bump and native music–international music, respectively. The scale used for the memory was the total number of songs that evoked memories, considering that 20 stimuli (5 stimuli x 4 levels) were used to calculate the means. For familiarity, we used a 3-point Likert scale and for valence and arousal, a 9-point Likert scale.

Regarding the OM× Age group effect, the OM factor showed an inverse relationship with valence, that is the effect of NAT, compared with INT, significantly increased the values in the group of older adults, *t*(14) = 4.15, *p* = .001, *d* = 1.58, while in the middle-aged adults this increase was found in the INT scores, although the differences between levels were non-significant, *t*(14) = -.309, *p* = .762. The between-groups comparisons within the same level revealed significant differences and a large effect size on NAT, with higher values in the older adults, *t*(28) = -2.18, *p* = .037, *d* = -.80, while on the INT level, the middle-aged adults evaluated the songs more positively, although the between-group differences were non-significant, *t*(28) = 1.24, *p* = .225. On the arousal variable, the OM factor showed no interaction. Both groups showed non-significant differences between NAT and INT and higher scores on NAT. These results suggest that the older adults were more sensitive (dependent variable, valence) to the songs from their youth compared with the later period, while they also evaluated the native songs more positively, while, in contrast, the middle-aged adults responded similarly regardless of the life stage and origin of the music.

As regards the number of memories, there was an increase in the responses from INT to NAT, although this was significant only in the older adults group, *t*(14) = 6.20, *p* < .001, *d* = 1.29, and not in the group of middle-aged adults, *t*(14) = 1.03, *p* = .320. The between-groups comparisons at the same level revealed significant differences and a large effect size on NAT, with higher scores in the older adults, *t*(28) = - 2.78, *p* = .009, *d* = -1.02. The differences on INT were non-significant, with the middle-aged adults scoring slightly higher, *t*(28) = 0.44, *p* = .662. In sum, the above shows that the older adults recalled significantly more memories with native music, although the characteristics of the study did not allow us to determine whether these memories were specific or general.

Finally, on the familiarity variable, the response levels were lower for INT compared with NAT, with the effect size being much higher in the group of older adults, *t*(14) = 7.75, *p* < .001, *d* = 2.28) compared to their middle-aged counterparts, *t*(14) = 2.66, *p* = .019, *d* = .48. The between-groups comparisons within each OM level revealed significant differences in both conditions, with scores being higher in the group of middle-aged adults and the effect being higher in INT, *t*(28) = 5.15, *p* < .001, *d* = 1.87 than in NAT, *t*(28) = 2.12, *p* = .043, *d* = 0.79. These results suggest, then, that the older adults were less familiar with the international music.

### Mood induction

The pre-post comparisons reveal that the task generated a significant increase in PA in both groups (middle-aged adults, *t*(14) = -3.20, *p* = .006, *d* = -0.28; older adults, *t*(14) = -5.29, *p* < .001, *d* = -0.69), with a higher effect size in the older adults. For NA, the pre-post comparisons showed a significant decrease (middle-aged adults, *t*(14) = 3.13, *p* = .007, *d* = 0.97; older adults, *t*(14) = 4.60, *p* < .001, *d* = 1.29), with a higher effect size in the older adults. Additionally, to verify the impact of the process of autobiographical memory retrieval on positive mood, we performed a hierarchical regression following the expression, PANAS Positive affect pretest = PANAS positive affect post-test+total number of memories retrieved. The complete model produces an *R*^*2*^ = .83 (*F* = 66.19, p < .001) with *B* = 1.01 for the value of positive affect in the pre-test (*ß* = 0.89, *t* = 11.24, p < .001, 95% CI 0.82–1.19) and with B = 0.03 for the total number of memories (*ß* = 0.16, *t* = 2.08, p = .04, 95% CI 0.82–1.19). These results suggest that the memory resulted in a slight significant increase in the post-test mood state.

## Discussion

Looking at the aims of our study, the experiment confirmed the idea that the RB life stage would have a greater effect on all the dependent variables in the study. This occurred in the group of older adults but not, however, in the group of middle-aged adults, who only showed a larger effect in the memory and familiarity variables. A similar effect was found for NAT, with a larger effect in all the dependent variables in the case of the older adults, with the exception of the arousal variable, where the between-level differences were non-significant in both age groups. Regarding the middle-aged adults, the differences between NAT and INT were only significant for familiarity. These findings suggest that, when this type of methodology is used with adults, the age groups should be clearly defined. As regards the analysis of the age effect on emotionality, the response appears to vary depending on the methodology used. In mood induction, the exposure to music led to an increase in positive affect and a decrease in negative affect, both being associated with a larger effect size in the older adults. With respect to the effect of the process of autobiographical memory retrieval on positive mood, the results suggest a slight, significant increase in mood state.

With regard to the improved effectiveness in memory retrieval compared with previous studies, and taking into account the results by condition, the older adults exhibited high recall levels (a mean of 80.67%) for the RB life stage, regardless of the origin of the music, and for native pop music (a mean of 78.67%), regardless of the life stage. In this sense, our conclusions are limited as the design of the experiment was incomplete because the memories were not recorded, for which reason E2 was designed with a focus on late adulthood and memory specificity. In sum, however, the design was considered appropriate for the research aims.

## Method, Experiment 2

### Participants

Thirty-five healthy older adults participated in the current study, *M* age = 67.25, *SD* = 3.47, range = 61–73; 62.9% female. General state of health, auditory functions, and years of academic education are shown in Table 1. In contrast to E1, to assess general mental health, we used Test Your Memory (TYM) [64], in its Spanish adaptation [65], *M* = 46.03, *SD* = 3.06, range = 40–50. The exclusion criteria were the same as in E1, with the exception of criterion (b). Depressive symptomatology was also taken into account, being assessed using the short depression item bank from the Patient-Reported Outcomes Measurement Information System (PROMIS^®^-Depression 4a) [66]. This version includes four items that evaluate participants’ negative affect over the last seven days, rated on a scale ranging from 1 = *never* to 5 = *always*, where the higher the score, the greater is the negative affect. Reliability for this scale is excellent (*α =* .96). In addition, participant age was limited to 73, as we found no international number one hits from before 1959 in the music charts we consulted. Apart from the participants selected for the study (N = 35), a total of 27 participants were excluded from the study; three who declined to take part in the second phase, five whose scores on the PROMIS–Depression test suggested they had depressive symptoms, and 19 who failed to reach the minimum score on the TYM. All participants gave written informed consent and were paid 35 € for their participation. Spanish was the first language of all the participants.

### Materials

#### Main study variables

We used the same scales as in E1, with the following exceptions:

*Musical stimuli*. The pop songs were selected from pop charts from the years between 1959 and 1991. A total of 64 excerpts were used (50% native popular music and 50% international popular music), each lasting 30 seconds. The selection criteria were the same as in E1, with the exception of the sources used to select the stimuli. In order to enhance song familiarity, we used data only from native charts, [54,55,57], most of which were official.

*Autobiographical memory*. In the experimental task, a dichotomous question was asked: memory evoked by the music, yes/no. Each memory was rated as *categorical*, *extended or specific*. To be coded as specific, the recalled event could not last longer than a day. Additionally, each memory was rated for whether it was positive, negative, or neutral. Two psychologists served as raters and independently scored the responses of all participants. Disagreements between ratings were resolved with a third evaluator.

### Procedure

The procedure was similar to that in E1, but with the following differences:

The tests were conducted between the months of February and November 2018. Seven experts took part in the screening phase and four researchers participated in the other tests.

Before beginning the experimental task (see S1 Fig, trial sequence), the participants were asked to relax and concentrate on their breathing for two minutes. The autobiographical memories were recorded for their subsequent coding. Participants were asked to provide a description with details of the autobiographical event, and the date and place where it occurred. They were given a time limit of five minutes, as in the study by El Haj et al. [49]. The task was conducted and the song excerpts were played on a desktop computer and without headphones. The participants took approximately 1h. 30min. to complete the experiment (Phase 2). In the experimental task, the mean length was 35.4 min., (*SD* = 0.13, range = 24 min. - 52.8 min).

### Data analysis

The data were organized with the aim of answering the following questions. What is the effect of musical cues from two consecutive life stages (LS), reminiscence bump and no reminiscence bump (RB-NORB), and different cultures (OM), native and international (NAT-INT) on memory specificity and positivity in older adults? What variables have most impact on the generation of both?

The data analyses were conducted by participant and by song. The original database was designed to have one entry per participant, enabling us to count the number of memories each participant generated. To assess the effect of LS and OM on valence, arousal, familiarity and autobiographical memory, we used a 2x2 mixed factor design, where LS (RB-NORB) and OM (NAT-INT) acted as within-subject factors. The Bonferroni’scorrection was used in the student’s t-tests.

However, our research questions in this second part of the study required a detailed analysis of the songs. To this end, the database was transposed, generating an entry for each song, meaning the database on 35 participants (with evaluations of 20 songs each), was now a database on 700 songs (evaluated by 35 participants). Using this data distribution, we calculated the percentage of positive memories associated with the valence of the songs, using two valence categories, low (≤ 6) and high (> 6).

In addition, to analyze the influence of valence, arousal, familiarity, LS, OM, and the effect of fatigue related to the number of stimuli (hereafter referred to as “number of songs or trials”) on the capacity to evoke any type of memory, we conducted a logistic regression with the memory/non-memory as a dependent variable. To examine the degree of association of valence, arousal, familiarity, LS and OM with the types of autobiographical memory, we conducted a multinomial logistic regression analysis, where the dependent variable comprised four categories, specific memory, categorical memory, extended memory and no memory (with the first being the reference category). The independent variables were valence, arousal, LS (two levels), OM (two levels), familiarity and number of songs. A total of 700 responses (20 songs x 35 participants) were used, of which 101 corresponded to specific memories (reference category), 184 categorical memories, 112 extended memories, 8 semantic memory associations, and 295 no memories. Emphasize that this analysis was done with an exploratory character. Taking into account that the data cannot be considered strictly independent. The statistical analyses were conducted using SPSS v.23 and the graphs in S3 and S4 Figs, were generated using the jitter option in the R ggplot2 package.

## Results

### Average percentages of memory and positivity

From a total of 700 trials, 397 autobiographical memories and 303 no memories (including 8 semantic associations) were generated. The average percentage of total positive memories, without taking “no memory” and “semantic association” into account was 88.52 (SD = 13.8), 8.40 (SD = 12.54) for neutral memory, and 3.08 (SD = 5.86) for negative. The average percentages of specific memory, total memory and standard deviation, according to LS × OM factors, are shown in Table 4 and S2 Fig. In this case, no memory and sematic association were considered.

**Table 4:** Average percentages of specific memory and total memory.

	Native songs		International songs	
	Reminiscence bump	No reminiscence bump	Reminiscence bump	No reminiscence bump
Specific Memory	25.14 (21.33)	12.00 (18.91)	13.71 (15.92)	6.86 (13.67)
Total memory	82.86 (18.87)	56.00 (28.61)	56.00 (28.20)	32.00 (25.76)

The values are expressed as average percentages (*SD*). The average percentages of specific memory and total memory, according to life stage (RB; NORB) × origin of the music (NAT; INT) factors, were calculated over a total of 5 trials.

The LS × OM comparisons of total memory revealed significant differences, with higher response levels for the RB-NAT condition, compared to RB-INT, *t*(34) = 5.62, *p* < .001, *d* = 1.45, and for NORB-SP, compared to NORB-INT, *t*(34) = 4.64, *p* < .001, *d* = 0.88, with the effect size being larger in the case of the RB life stage.

#### Positive music ratings associated with positive memories

The percentage of songs rated with high (>6) and low (≤6) valence (9-point Likert scale) associated with positive memories, without considering the no memory and semantic association categories was 78.82 and 10.84, respectively. These results suggest a positive relationship between the valence scores for the songs and the positivity of the memories recalled.

### Effect of life stage and origin of the music

The results of the repeated measures ANOVA (Table 5) revealed, at within-subject level, a significant main effect of LS on all the dependent variable scores except arousal. All the scores were higher in the RB condition. Similarly, OM had a significant effect on all the variables except arousal (see mean scores in Table 6). Regarding the LS×OM effect, the results revealed two significant interactions with medium-low effect sizes (Table 5). In specific memory, the LS effect generated a significant increase in the number of memories corresponding to the RB period, compared with NORB, while the OM effect generated a significant increase in NAT compared with INT, where the comparison of scores between the LS levels with respect to OM showed significant differences and a larger effect size for the RB level, *t*(34) = 3.26, *p* = .003, *d* = 0.61, while no significant differences were found for NORB, *t*(34) = 1.60, *p* = .119. It appears, therefore, that the native songs had a greater effect in the RB life period, while in the NORB period, the participants exhibited no significant differences in memory recall depending on the origin of the music. In valence, the LS effect generated higher scores in RB compared to NORB, while the OM music effect gave rise to higher scores on NAT compared to INT. The comparisons of scores between the LS levels with respect to OM revealed significant differences and a larger effect in the NORB life stage, *t*(34) = 4.25, *p* < .001, *d* = 0.71, while, in contrast, for RB the differences were non-significant, *t*(34) = 1.88, *p* = .068. In short, the results suggest that the participants evaluated the stimuli related to the RB stage more positively, compared to NORB, regardless of the origin of the music, while in the NORB period, the native pop songs were scored higher.

**Table 5:** Repeated measures analysis.

		Life stage		Origin of the music		Life stage *Origin of the music	
	*M*(*SD*)	*F*	ηp²	*F*	ηp²	*F*	ηp²
Total Memory	11.34(3.62)	49.74***	.59	45.08***	.57	.18	.01
Specificity	2.88(2.61)	19.83***	.37	7.64**	.18	4.27*	.11
Familiarity	2.34(.37)	61.90***	.64	67.62***	.66	2.48	.07
Valence	7.12(.89)	49.27***	.59	14.50**	.30	4.99*	.13
Arousal	4.95(1.68)	.01	.00	.76	.02	.05	.002

***p < .001,

**p < .01,

*p < .05. The means refer to the total of each measure. The mean values are expressed as *M*(*SD*). The life stage or period and origin of the music comprise the levels of reminiscence bump—no reminiscence bump and native music–international music, respectively. The scale used for the total memory (sum of the memory categories: specific, categorical and extended) was the total number of songs that evoked memories by condition, for specificity was the number of songs that evoked specific memories. For familiarity, we used a 3-point Likert scale and for valence and arousal, a 9-point Likert scale.

**Table 6:** Mean scores for the variables of total memory, specific memory, familiarity, valence, arousal in relation to life stage (reminiscence bump- no reminiscence bump; RB-NORB) and origin of the music (native music–international music).

	Reminiscence bump	No reminiscence bump	Native music	International music
	*M(SD)*	*M(SD)*	*M(SD)*	*M(SD)*
Total memory	6.94(1.94)	4.40(2.25)	6.94(1.83)	4.40(2.39)
Specific memory	1.94(1.57)	0.94(1.35)	1.86(1.85)	1.03(1.25)
Familiarity	2.53(0.37)	2.15(0.41)	2.55(0.28)	2.15(0.41)
Valence	7.52(0.91)	6.73(0.99)	7.41(0.87)	6.84(1.12)
Arousal	4.96(1.81)	4.94(1.64)	5.02(1.78)	4.89(1.68)

The mean values are expressed as *M*(*SD*). The scale used for the total memory (sum of the memory categories: specific, categorical and extended) was the total number of songs that generated memories by condition, taking into account these are within-subject comparisons and the memories described refer to sets of 10. Thus, on the one hand, they are compared according to whether they belong to the reminiscence bump and, on the other, depending on whether they are native or international. As an example, the reminiscence bump comprises 5 native and 5 international songs. Specificity was the number of songs that evoked specific memories by condition. For familiarity, we used a 3-point Likert scale. For arousal and valence, a 9-point Likert scale.

### Relationship between types of memory and valence, arousal, familiarity, life stage, origin of the music and number of stimuli in the experimental task

To analyse the influence of valence, activation, familiarity, life period, origin of the music and the number of songs on the capacity to evoke or not any kind of memory, we have made a logistic regression with the memory/non-memory as a dependent variable. The results of the goodness of fit test showed an association between the independent variables and the dependent variable, χ² = 307.92 (6), p < .001. The Cox and Snell R Square was .356 and Nagelkerke’s R Square was .477. The model correctly classified 80.1% of the cases. All independent variables show significant differences (Wald’s test) in the prediction of the recall variable (see S1 Table).

A one-unit increase in the variables of valence, arousal and familiarity, compared to the non-increase of one unit in those variables, if the other variables remain constant, would increase the odds ratio (OR) of the response being any type of memory. As regards the factors of life stage and nationality of the music, the songs from the NORB compared with those from the RB would decrease the OR of generating a memory, and the native songs compared to the international songs would increase the OR of generating a memory. Regarding the number of songs, the results showed a one-unit increase would decrease the OR of generating memories. These findings suggest that all the independent variables are solid predictors to discriminate between no memory and memory, with familiarity being that which produces the largest effect.

For the analysis of the types of memory (specific, categorical, extended and no memory) and the selected independent variables, we used multinomial logistic regression, with specific memory as the reference category. The results of the goodness of fit test showed an association between the independent variables and the dependent variable, χ² = 325.18 (18), p < .001. The Cox and Snell R Square was .372 and Nagelkerke’s R Square was .402. In addition, the model correctly classified 52.1% of the cases (specific memory 1%, categorical memory 60.9%, extended memory 4.5%, and no memory 81.5%). The categories of categorical and extended memories showed no significant relationship (Wald’s test) with the independent variables, although a quasi-significant relationship was found between familiarity and the extended memory category (Table 7). All the relationships with the no memory category were significant, except for that of the number of trials, which was only quasi-significant. Regarding valence, arousal and familiarity, a one-unit increase in these variables, compared to the non-increase of one unit in those variables, if the other variables remained constant, would decrease the OR of the response being a no memory. The LS and OM factors showed a similar effect in relation to the no memory category. The songs from the RB period, compared with the non-RB songs, decreased the OR of generating a no memory, and the native songs, compared with the international songs, increased the OR of evoking a memory. These findings suggest that the independent variables used in the study are good predictors of specific memory with relation to no memory but not in relation to categorical and extended memories.

**Table 7:** Multinomial logistic regression.

Memory types	Variables	B	SE	Wald	sig	Exp(B)	95%CI
CATEGORICAL	Intercept	2.91	0.96	9.15	.002		
	Valence	-0.16	0.11	2.36	.124	0.85	0.69–1.05
	Arousal	0.00	0.05	0.00	.959	1.00	0.91–1.10
	Familiarity	-0.17	0.30	0.35	.574	0.85	0.47–1.52
	Life stage	-0.39	0.27	2.14	.143	0.68	0.40–1.14
	Origin of the music	-0.30	0.26	1.36	.243	0.74	0.44–1.23
	Sequence of trials	-0.01	0.02	0.46	.497	0.98	0.94–1.03
EXTENDED	Intercept	2.16	1.04	4.30	.038		
	Valence	-0.04	0.12	0.09	.767	0.96	0.76–1.22
	Arousal	0.03	0.05	0.34	.561	1.03	0.93–1.15
	Familiarity	-0.59	0.31	3.50	.061	0.55	0.30–1.03
	Life stage	-0.04	0.30	0.02	.890	0.96	0.54–1.72
	Origin of the music	0.08	0.29	0.08	.774	1.09	0.61–1.93
	Sequence of trials	-0.04	0.02	2.47	.116	0.96	0.92–1.01
NO MEMORY	Intercept	8.73	0.96	83.26	< .001		
	Valence	-0.31	0.11	8.04	.005	0.74	0.59-.91
	Arousal	-0.13	0.05	5.60	.018	0.88	0.79-.98
	Familiarity	-1.79	0.29	38.32	< .001	0.17	0.09-.29
	Life stage	-0.93	0.28	10.92	.001	0.40	0.23-.69
	Origin of the music	-0.88	0.28	10.06	.002	0.42	0.24-.71
	Sequence of trials	0.04	0.02	3.10	.078	1.04	0.99–1.09

The reference variable is specific memory. Life stage comprises the levels of reminiscence bump–no reminiscence bump. Origin of music comprises the levels of native music–international music. Sequence of trials / number of songs indicates the effect of fatigue related to the number of stimuli. For familiarity, we used a 3-point Likert scale and for arousal and valence, a 9-point Likert scale. Of 700 trials, 101 generated specific memories, 184 categorical memories, 112 extended memories, 303 no memories (including 8 semantic associations).

For emotionality, the graphical distribution of scores, as shown in S3 Fig, reveals most of the values associated with the valence of the memory types are situated on the positive side of the scale (> 5), while, for no memory, the values are distributed across all the levels, although the greatest number are situated in the mid-and high parts of the scale. For arousal, the distribution is also similar across the memory types, moderately associated with high arousal (> 5). In contrast, the values for no memory exhibit a slight increase in the mid- and low part of the scale (≤ 5).

The distribution of familiarity scores (S4 Fig), in relation to valence and memory types, shows they are mostly situated in the quadrant corresponding to high familiarity (≥2) and high valence (>5) in all the categories. The distribution of the no memories are mainly situated around the middle of the scale for both variables, although there are scores distributed across all the quadrants. These results suggest a positive relationship between valence, familiarity and memory (see S3 and S4 Figs).

Finally, the results show that the number of songs used in the task has no impact on the increase in no memories compared to specific memories.

### Mood induction

The pre-post mood state assessment was conducted with a dependent measures design (t test), using measures from the PANAS (PA and NA subscales). The results of the pre-post comparisons revealed a significant increase in PA with a medium-low effect size, *t*(34) = -2.11, *p* = .042, *d* = -0.30 and a significant decrease in NA with a large effect size, *t*(34) = 4.72, *p* < .001, *d* = 0.80.

## Discussion

As in E1, the results showed a higher effect of RB and NAT in all the dependent variables with the exception of arousal, where the differences between life stages and types of music were non-significant. In addition, the task generated a positive effect on mood state, increasing positive affect and decreasing negative affect. As regards the effectiveness of the design used for memory recall, the mean specificity percentage, taking into account all the experimental conditions, was relatively low (14.43%), although if we focus on the results of the combination of RB-NAT cues, the level could be considered satisfactory (25.14%). Following our hypothesis, the relationship between the emotional evaluation (valence) of the music and memory positivity was positive. The analysis of the relationships between the types of memory and the characteristics that impact their generation, it appears that the independent variables selected are not good predictors in differentiating between such types. However, they are good predictors of no memory. It is worth noting that familiarity was the variable that generated the largest effect when predicting memory in general. In addition, we controlled for the effect of the number of song excerpts used in the experiment on memory retrieval, with the results showing a non significant effect. In contrast, the results of the logistic regression with the memory/non-memory as a dependent variable revealed a significant effect. This is a novel contribution in this field of study. In short, considering the research aims, the results obtained can be regarded as satisfactory.

## General discussion

Our first aim was to determine the effect of age and pop music of different origin from two consecutive life periods on emotionality, autobiographical memory and song familiarity. In E1, coinciding with previous studies [28], and confirming our first hypothesis, the results showed a significant greater effect of the RB life stage on all the dependent variables, in the case of the older adults but not in the case of their middle-aged counterparts, where the comparisons between life periods in the arousal and valence variables were non-significant. It should be noted that the study conducted by Schulkind et al. [28] included neither the arousal variable nor a group of middle-aged adults. Regarding the factor of age, at between-subjects level, the results of the ANOVA revealed a significant effect only on familiarity, where the group of middle-aged adults scored higher on responses on all levels. These findings are similar to those in the work by Bartlett et al. [47] . It is also worth noting that some previous studies [22] comparing responses between young (*M* age = 21.4, SD = 2.77) and older adults (*M* age = 70.31, *SD* = 7.27) failed to find differences. This might suggest that familiarity with popular music shows a peak related to middle adulthood. Despite the difference in the level of popularity of the songs between studies, which conditions any possible comparisons, given the methodology used, this finding appears to be of interest.

As regards the emotionality evoked by the music (valence), the Age group x LS interactions showed significant differences between the two life periods only in the older adults group, where an increase was found in the RB life stage. On the other hand, the between-groups differences within the same life period were non-significant, although the older adults rated the RB songs more positively and the middle-aged adults gave more positive ratings to the NORB songs. These findings give rise to various interpretations. Firstly, the results for the older adults comparing the RB life stage with the NORB period appear to coincide with the socioemotional selectivity theory [see, 36 for a review], which posits a relativity bias that emerges in late adulthood. Secondly, focusing only on the NORB life stage, we find higher positivity ratings among the middle-aged adults, although these differences are non-significant. This would seem to be partially in line with the study by Schweizer et al. [46] , in which the authors, using a different methodology and cues, report a progressive decline in positivity as individuals grow older. A further interpretation might be the existence of a dependent variable effect of the type of stimuli and methodology used in the studies, as appears to be our case. It is worth noting that, in contrast to our study, where the group of older adults in E1 included no participants with a university education, most participants in these studies have a university degree, a variable to be considered in this field of research [67].

The second aim was to study the effect of musical culture through the origin of the music. The ANOVA results revealed a significant main effect of native popular music on all the dependent variables except arousal. It should be underlined that in the group of middle-aged adults, significant differences between NAT and INT music were only found for familiarity, with higher levels with regard to NAT. Regarding familiarity, these results are similar to the findings for the LS factor, although in this case the effect size between the different levels was much larger, in the case of the older adults. This coincides with the findings of Demorest et al. [43] and Morrison et al. [44] , who reported that more culturally familiar or closer music is remembered more successfully. An important difference between these two cited works and the current one is that the international pop music used was not strictly speaking from a different culture, but is rather part of a general Western cultural context. This, together with much larger effect size in the older adults, should be interpreted with caution, as another interpretation of these findings may lie in the fact that access to music in Spain at the beginning of the 1950s was primarily in the form of live performance. It should also be added that until the end of the 1950s, pop music was not commonly played on the radio [68], conditioning the access of older adults to international music at that time. This might suggest, in addition to cultural influence, a simple effect of exposure to music.

Continuing with the OM factor, the conclusions that can be drawn regarding the subjective evaluation of the valence of the songs are similar to those for the LS factor with respect to the positivity effect in the older adults. On the other hand, focused on the group of older adults and taking into account our findings on the familiarity variable, the above results are in line with conclusions reported in previous studies [69], that is, affective responses to music are more determined by cultural tradition than by the inherent qualities of the music. Complementarily, authors, such as Sloboda et al. [8] , have conducted literature reviews finding that emotional responses to music are linked to everyday activities, suggesting that on occasions these responses may be influenced by interaction between cognitive and affective processes. In this sense, in the current studies, the older adults were found to give more positive ratings to native or culturally more familiar pop music, while the middle-aged adults showed no differences in their affective evaluations depending on familiarity, suggesting that affective responses are also conditioned by a cognitive component or a learning factor.

The first aim of E2 was to confirm the findings of E1 on the effect of RB and NAT, focusing on this occasion on a group of older adults. The second aim was to attempt to enhance the effectiveness of the procedure with regard to the positivity evoked by the songs and the specificity of the autobiographical memories retrieved. As in E1, the ANOVA results confirmed a significant main effect of LS and OM on all the dependent variables (higher RB and NAT scores) except arousal. Given the influence of familiarity on memory in this study context, it is worth noting with regard to the RB effect on song familiarity that the findings obtained contrast with those of the study by Platz et al. [24] (Experiment 1), in which no main effect between different decades (1930–2010) was found. In contrast to the present study, the authors used age-specific songs associated with seven periods, corresponding to when the participants were aged between 5 and 82 years (*M* age of sample = 67.1, *SD* = 6.8, range = 52–82). A potential explanation for this difference in results is that their study used fewer songs per period and participant, around just two, while in our study, 10 were used, thus increasing the likelihood of the stimuli being more familiar. In addition, it is worth remembering what we previously mentioned about access to music in Spain until the end of the 1950s.

Furthermore, the RBxOM interactions showed a medium-size effect on valence and memory specificity, broadly suggesting that the combination of the effect of the RB and NAT conditions generates an increase on both dependent variables. As for the effectiveness of the procedures, despite the relatively low specific memory recall (14.43%), taking into account the overall experimental conditions and focusing on the results of the RB-NAT combination (25.14% specific memory), the levels can be considered satisfactory. In the study by Schulkind et al. [28] , of the memories generated over 60 trials per participant by the group of older adults (*M* age = 67.5, *SD* = 2.2), only 4% referred to specific events (in the block corresponding to the RB, it was 8%). It should be noted that, in comparison to the present study, the number of songs was much higher, which might arguably have generated fatigue in the participants, as demonstrated in studies using visual stimuli [34,35].

Continuing to look at results on memory specificity in other studies, Platz et al. [24] , in the age decades when the participants were aged 5–14 years and 15–24 years, specificity was 8.5% and 7.4%, respectively. Regarding total memories, in the present study, the participants retrieved a mean number of 11.34 memories over 20 trials, and for the RB life stage and native music, the mean was 82.86% over 5 trials. This is in line with the findings of the study by Ford et al. [22] , in which participants retrieved 76% over 30 trials. In conclusion, these data, coinciding with the work of other authors [22,24,25,28] confirm the potential of popular music, and especially of this methodology, to generate autobiographic memories.

With regard to the relationships between the characteristics of the musical cues, the emotions they evoke and the types of autobiographical memories, the results of the multinomial logistic regression suggest that emotionality (valence-arousal) is not a conclusive determinant of the different types of memory recalled. However, it does appear to be a robust predictor of no memory compared to memory, something which few studies have reported. Furthermore, confirming our hypothesis, the results show a positive relationship between the valence generated by the songs and the total number of positive memories evoked, considering that the percentages of songs rated with high valence (>6) associated with positive memories, without accounting for no memory and semantic association, was 78.82. With reference to the arousal variables, as can be seen in S3 Fig, the memory scores are distributed across the entire scale (9-point Likert-type), which is consistent with the findings of Schulkind et al. [29] . As for the valence generated by the songs and the positivity of the memories, the results confirm the theories on mood and memory posited by Bower [70] , which suggest that emotion-loaded cues facilitate retrieval of memories of a similar tone.

The regression analyses revealed no significant relationship between familiarity with the music and the different types of memory (categorical and extended), although they did show a significant difference in the relationship between this variable and no memory and specific memory. Moreover, of all the independent variables analyzed, these produced the greatest effect. In addition, as shown in S4 Fig, the familiarity scores with relation to memory are mainly situated in the mid- and high part of the scale. This suggests that the familiarity variable is a good predictor of memory and its associated positivity, with relation to no memory. This is consistent with the study by Ford et al. [22] , the results of which revealed a positive correlation between memory positivity and song familiarity, while no increase in specificity was found related to familiarity.

One of the novel contributions of our study is related to the analysis of the effect of fatigue related to the number of stimuli used in the memory retrieval task, where it was found that with regard to specific memory, the number of trials does not increase the number of no memories. In contrast, the results of the logistic regression with the memory/no memory as a dependent variable revealed a significant effect. This is a novel contribution in this field of study. The participants took a mean time of 35.4 min, to complete the task in question, suggesting, with regard to specific memory, that the design and number of songs used was adequate for the aims of our research.

Finally, with regard to the mood induction, after doing the experimental task, in both studies the participants showed an increase in positive affect and a decrease in negative affect, results that go beyond those reported by Janata et al. [25], where a decline only in negative affect was found. This might be because the participants in their study were younger (*M* = 20.6, *SD* = 1.8) and the stimuli were selected from among the Billboard Top 100, which might mean some of the music was less popular. It is worth remembering that the present work used songs that had reached number 1 in the sales charts. Additionally (E1), the intention was to determine to what extent the process of autobiographical memory retrieval impacts on mood state. The results suggest a slight, significant association with an enhancement of mood state. We should mention certain differences with the studies cited herein, which limit our conclusions and possible comparisons. First, the number of participants in the two experiments was relatively small. The number of specific memories used in the regression analysis in E2 was also small (101), if we compare it with the number, for example, of categorical memories (184) or no memories (295). It should also be noted that the familiarity scale used only comprised three levels, which might have affected the precision of the results. It should also be noted that the design might be considered incomplete, given that the evaluation of the emotional characteristics of the memory cues was not “external and objective”, in the sense that the participants themselves were responsible for the ratings. Furthermore, participants were not explained the difference between an emotion perceived and an emotion felt. In other words, the participants’ evaluations were arguably a mixture of the emotion expressed in the music and their own emotional response, as suggested by some authors [67]. In this respect, it is worth noting that our study used a pre-post test measure of mood state, and that the results related to positive memory are similar to those of other studies that used an “external measurement” of the songs [29] , showing that most of the memories generated, regardless of the valence of the stimuli, were positive. Another consideration is that English is not the native language of the participants, who also presented different related academic levels. This fact, unlike musical comprehension, which allows multiple interpretations, could influence the linguistic comprehension of songs, a factor relatively related to familiarity in this study context [71]. Finally, the songs used were not chosen by the participants, which notably limits familiarity with the music.

In summary, our study confirms the RB effect in older adults reported in previous works. It also broadens knowledge on the characteristics that influence the interaction of emotion-memory-music, including the subjective cultural component. Moreover, the results show that this methodology is an effective tool to generate positive mood states and enhance autobiographical memory recall.

## Supporting information

S1 Fig Trial sequence. (A) Relaxation. (B) Explanation. (C) Instruction. (D) Black screen. (E) Song excerpts, 20 in total, lasting 30,000 ms (F) Question on emotions scored on the SAM scale. (G) Question on song familiarity. (H) Question on the memory, dichotomous response. (I) Screen prior to description of memory (Study 2). (J) Black screen. (K) Recording of memory, blue screen. (L) Optional pause before the next trial.
(TIF)

S2 Fig Average percentages and standard deviation of total and specific memory. Average percentages by life stage (LS) = Reminiscence bump (RB), no reminiscence bump (NORB) and origin of the music (OM) = native (NAT) and international (INT) songs. The LS×OM comparisons of both memory types were conducted using a paired t test, p < .001***, p < .01**, p < .05*, ns = non significant.
(TIF)

S3 Fig Distribution of valence and arousal generated by the songs in relation to the different types of memory. Of 700 trials, 101 evoked specific memories, 184 categorical memories, 112 extended memories and 303 no memories (including 8 semantic associations).
(TIF)

S4 Fig Distribution of valence generated by the songs and song familiarity in relation to memory and no memory. Of 700 trials, 397 evoked memories and 303 no memories (including 8 semantic associations).
(TIF)

S1 Table Logistic regression model to classify between memory and no-memory. (PDF)

S1 Appendix (PDF)

S2 Appendix Experiment 1, ANOVA and t-test database. (XLSX)

S3 Appendix Experiment2, ANOVA and t-test database. (XLSX)

S4 Appendix Experiment 2, regression database. (XLSX)
