# Supplementary figures and images for: Autobiographical emotional induction in older people through popular songs: Effect of reminiscence bump and enculturation
Source: PLoS One. 2020 Sep 2;15(9):e0238434. doi: 10.1371/journal.pone.0238434 (PMC7467226; doi:10.1371/journal.pone.0238434)

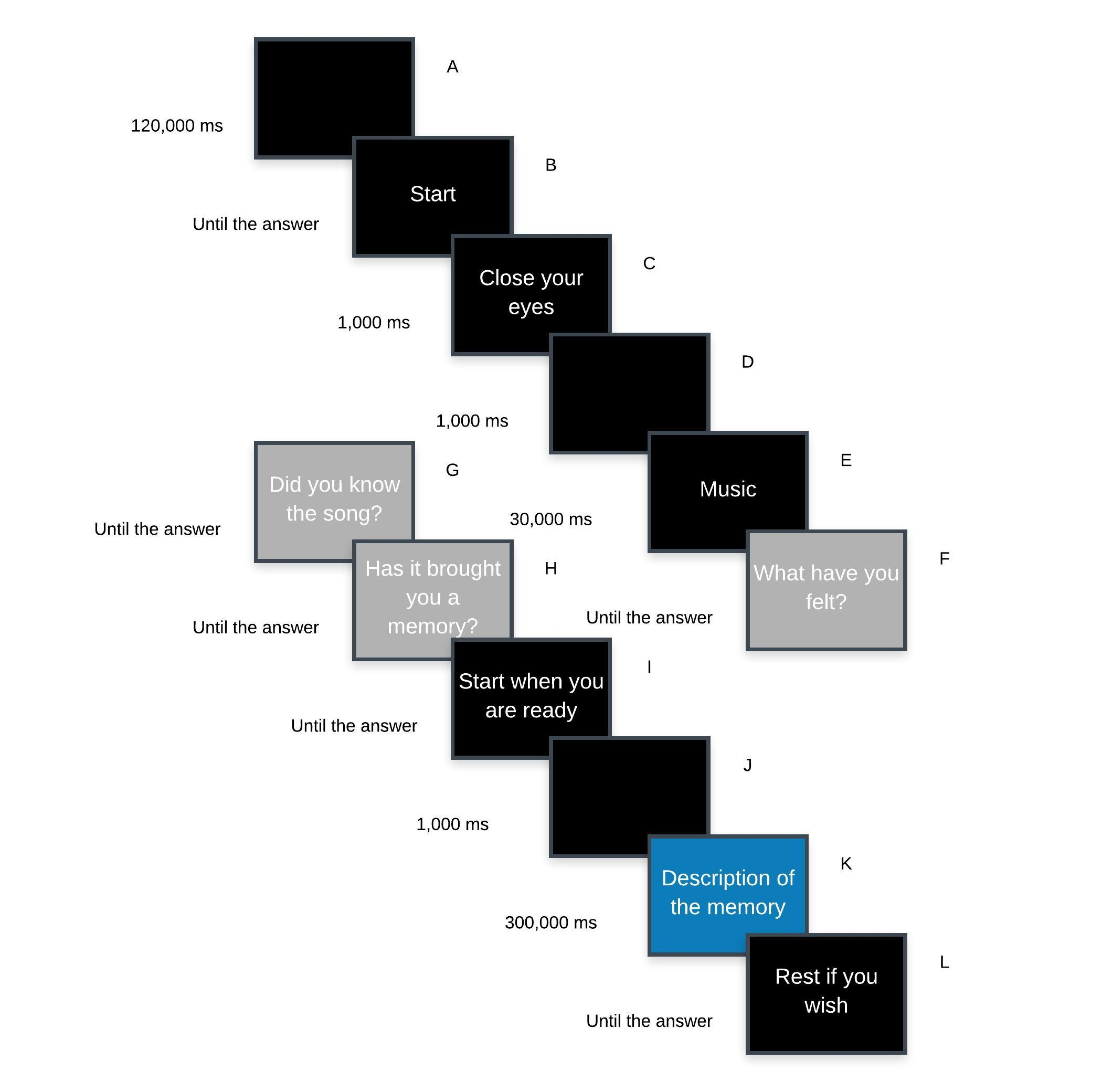

Supplement: S1 Fig — (A) Relaxation. (B) Explanation. (C) Instruction. (D) Black screen. (E) Song excerpts, 20 in total, lasting 30,000 ms (F) Question on emotions scored on the SAM scale. (G) Question on song familiarity. (H) Question on the memory, dichotomous response. (I) Screen prior to description of memory (Study 2). (J) Black screen. (K) Recording of memory, blue screen. (L) Optional pause before the next trial. (TIF) [file pone.0238434.s001.tif]

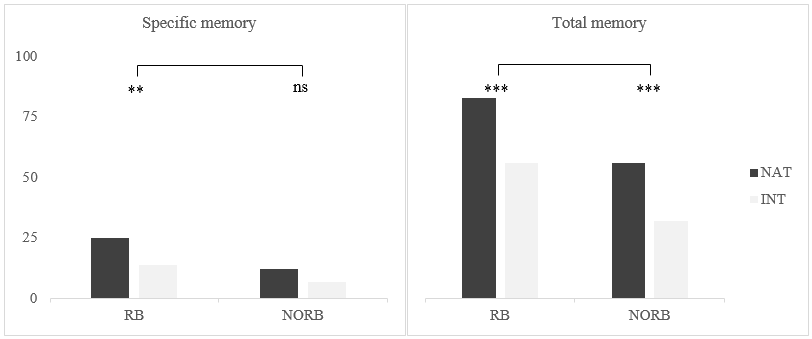

Supplement: S2 Fig — Average percentages by life stage (LS) = Reminiscence bump (RB), no reminiscence bump (NORB) and origin of the music (OM) = native (NAT) and international (INT) songs. The LS×OM comparisons of both memory types were conducted using a paired t test, p < .001***, p < .01**, p < .05*, ns = non significant. (TIF) [file pone.0238434.s002.tif]

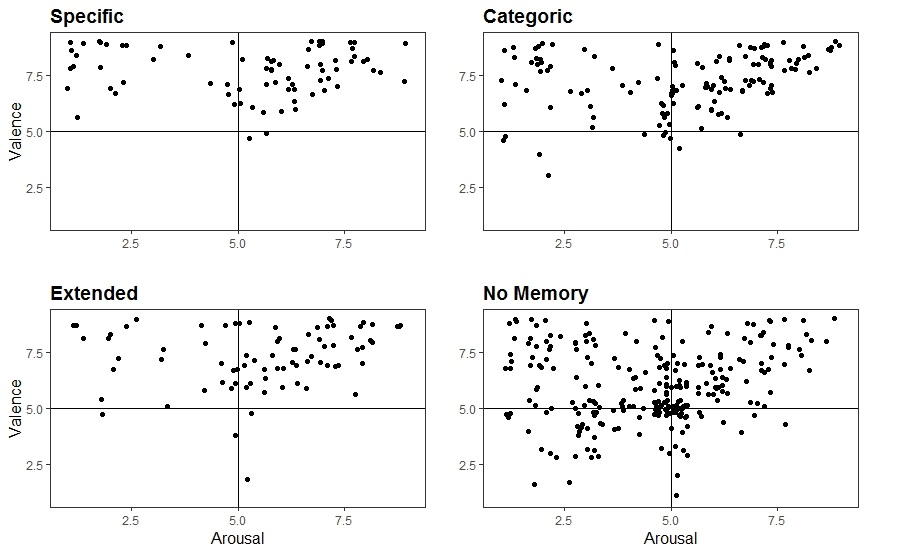

Supplement: S3 Fig — Of 700 trials, 101 evoked specific memories, 184 categorical memories, 112 extended memories and 303 no memories (including 8 semantic associations). (TIF) [file pone.0238434.s003.tif]

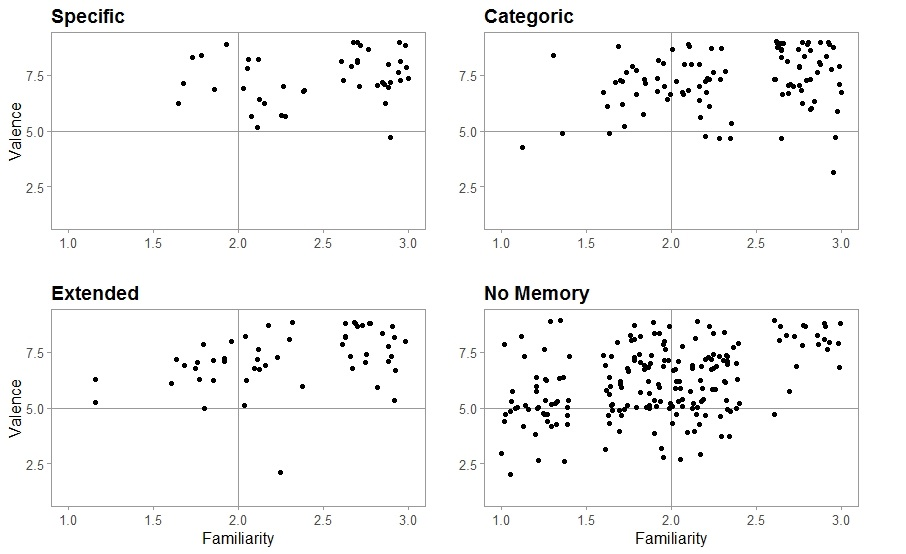

Supplement: S4 Fig — Of 700 trials, 397 evoked memories and 303 no memories (including 8 semantic associations). (TIF) [file pone.0238434.s004.tif]
