# Supplementary material for: Autobiographical emotional induction in older people through popular songs: Effect of reminiscence bump and enculturation
Source: PLoS One. 2020 Sep 2;15(9):e0238434. doi: 10.1371/journal.pone.0238434 (PMC7467226; doi:10.1371/journal.pone.0238434)
Supplement: S1 Table — (PDF) [file pone.0238434.s005.pdf]

S1 Table

Logistic regression.

	Variables	B	SE	Wald	sig	Exp(B)	95%CI
ANY KIND OF MEMORY	INT	-4.01	0.60	46.90	<.001		
	VAL	0.21	0.07	10.71	.001	1.24	1.08-1.42
	ARS	0.14	0.42	10.78	.001	1.15	1.06-1.25
	FAM	1.52	0.18	74.65	<.001	4.60	3.25-6.50
	LS	-0.73	0.20	13.17	<.001	0.48	0.33-0.72
	OM	-0.75	0.20	14.11	<.001	0.47	0.32-0.70
	SEQ	-0.06	0.02	11.94	.001	0.94	0.91-0.97

Note. INT = Intercept, VAL = valence, ARS = arousal, FAM = familiarity, LS = Life stage (reminiscence bump – no reminiscence bump), OM = Origin of music (native-international), SEQ = sequence of trials / number of songs. The scale used for familiarity was a Likert scale 1-3 and for valence and arousal a Likert scale 1-9. Of 700 trials, 397 generated any kind of memory and 303 no memories (including 8 semantic associations). Study 2.
