# Supplementary material for: Autobiographical emotional induction in older people through popular songs: Effect of reminiscence bump and enculturation
Source: PLoS One. 2020 Sep 2;15(9):e0238434. doi: 10.1371/journal.pone.0238434 (PMC7467226; doi:10.1371/journal.pone.0238434)
Supplement: S1 Appendix — (PDF) [file pone.0238434.s006.pdf]

S1 Appendix

Musical stimuli Study 1

Year	Spanish songs<br>Artist - song	International songs<br>Artist - song
1952	Miguel de Molina - La bien pagá	Kay Starr - The Wheel of fortune
1953	Luis Mariano - Doce cascabeles	Perry Como – Don't let the stars get in your eyes
1954	Concha Piquer En tierra extraña	The Crew Cuts - Sh Boom Sh Boom
1955	Luis Mariano - Violetas imperiales	Bill Haley - Rock around the clock
1956	Joselito - Campanera	Elvis Presley - Don't be cruel
1957	Gracia Montes - Será una rosa	Jimmie Rodgers - Honeycomb
1958	Las Hermanas Fleta -.El chacacha del tren	Domenico Modugno - Nel blu dipinto di blu
1959	José Luis - Mariquilla bonita	Bobby Darin - Mack the Knife
1960	Gloria Lasso - La Canción De Orfeo	Bob Azzam - Mustapha
1961	Dúo Dinámico - Quince años tiene mi amor	The Marcels - Blue Moon
1962	Marisol - Tómbola	Paul Anka - Steel guitar and a glass of wine
1963	Enrique Guzmán – Dame felicidad	Louis Armstrong - Hello Dolly
1964	Los Pekenikes - Los cuatro muleros	The Beatles - She love you
1965	Los Sirex - Si yo tuviera una escoba	The Beatles - Yesterday
1966	Los Brincos - Mejor	Frank Sinatra - Strangers in The Night
1967	Los bravos - La motocicleta	The Beach Boys - Good Vibrations
1968	Massiel - La, la, la	Simon & Garfunkel - Mrs. Robinson
1969	Karina - Las flechas del amor	The Archies - Sugar sugar
1970	Los Diablos - Un rayo de sol	Venus - Shocking Blue
1971	Peret Borriquito	Rolling Stones - Brown sugar
1972	Nino Bravo - Un beso y una flor	Don McLean - American pie
1973	Camilo Sesto - Amor amar	Roberta Flack - Killing me softly with his song
1974	Juan Pardo - Conversaciones Conmigo Mismo	Bob Marley - No Women No Cry
1975	Desmadre 75 - Saca el Güisqui, Cheli	Barry White - You're my first my last my everything
1976	Jarcha - Libertad sin ira	Santana - Europa
1977	Pablo Abraira – Gavilán o paloma	Eagles - Hotel California
1978	Los Pecos Esperanzas	Bee Gees - Night fever
1979	Tequila - Me vuelvo loco	The Knack - My Sharona
1980	Los Pecos - Háblame de ti	Queen - Crazy little thing called love
1981	Maria Jesus - El baile de los pajaritos	Kim Carnes - Bette Davis eyes
1982	Mecano - Me cole en una fiesta	Lime - Babe we're gonna love tonight
1983	Miguel Ríos - El rock de una noche de verano	The Police - Every breath you take
1984	La unión - Hombre lobo en Paris	Michael Jackson - Thriller
1985	Alaska y Dinarama - Cómo pudiste hacerme esto a mí	Dire Straits - Walk of life
1986	Ana Belen y Victor Manuel - La puerta de Alcalá	The Bangles - Walk like an Egyptian
1987	Radio Futura - La negra flor	George Michael - Faith

1988	Dhuncan Dhu - Una calle de París	U2 - Desire
1989	Camaron De La Isla - Soy gitano	Kaoma - Lambada
1990	Radio Futura - Veneno en la piel	MC Hammer - U can't touch this
1991	Héroes del Silencio - Entre dos tierras	Bryan adams - Everything I do
1992	Celtas cortos – El ritmo del mar	Nirvana - Come As You Are
1993	El Ultimo de la Fila - Como Un Burro amarrado	UB40 - Can't help falling in love
1994	Amistades Peligrosas - Me haces tanto bien	Ace of Base - The Sign
1995	Luz Casal - Entre mis recuerdos	Ini Kamoze - Here Comes The Hotstepper
1996	Los del Rio - La Macarena	Lemon Tree - Fools Garden
1997	Jarabe De Palo - La Flaca	Hanson - Mmmbop
1998	Alejandro Sanz - Corazon partio	Cher - Believe
1999	Joaquin Sabina - 19 días Y 500 noches	Ricky Martin - Livin' la vida loca
2000	Estopa - Como Camaron	Santana - Maria
2001	Manu Chao - Me Gustas Tu	Kylie Minogue - Can't get you out of my Head
2002	Las kepchup - Asereje	Shakira - Whenever, Wherever
2003	La Cabra Mecanica - No me llames iluso	Black Eyed Peas - Where Is The Love
2004	Antonio Orozco - Quiero ser	O Zone - Dragostea din tei
2005	Amaral - Días De Verano	Maroon 5 - She Will Be Loved
2006	Fito y Fitipaldis - Por la boca vive el pez	Gnarls Barkley - Crazy
2007	La Quinta Estación - Me muero por besarte	Alicia Keys - No one
2008	Chambao - Papeles mojados	Coldplay - Viva La Vida
2009	Macaco - Moving	Jason Mraz - I'm Yours
2010	Maldita Nerea - El secreto de las tortugas	Shakira – Waka
2011	Juan Magan - Bailando por ahí	Gotye - Somebody That I Used To Know
2012	Cali & El Dandee - Yo te esperaré	Michel Teló - Ai se eu te pego
2013	Pablo alborán – El Beso	Avicii – Wake me up
2014	Malu – A prueba de ti	Pharrell Williams - Happy
2015	Alvaro soler – El mismo sol	OMI - Cheerleader
2016	Pablo López – Tu enemigo	Justin Bieber – Love Yourself
2017	Leiva – La lluvia en los zapatos	Ed Sheeran – Shape of you

Musical stimuli Study 2

	Spanish songs	International songs
Year	Artist - song	Artist - song
1959	José Luis - Mariquilla bonita	Paul Anka- Diana
1960	Sara Montiel - Ola, Ola, Ola	Jacqueline Boyer - Tom Pillibi
1961	Carmen Sevilla - Eres Diferente	The brothers four - The green leaves of summer
1962	Hermanos Rigual - Cuando calienta el sol	Gilbert becaud - Et maintenant
1963	Enrique Guzmán – Dame felicidad	Rita Pavone - Cuore
1964	Dúo dinámico – Amor de verano	The Beatles - A hard day's night
1965	Los Brincos - Flamenco	France Gall - Poupee de cire
1966	Raphael - Yo soy aquel	Frank Sinatra - Strangers in The Night
1967	Los Bravos - Los Chicos con las Chicas	The Beach Boys - Good Vibrations
1968	Massiel - La, la, la	Tom Jones - Delilah
1969	Karina - Las flechas del amor	The Archies - Sugar
1970	Los Diablos - Un rayo de sol	Venus - Shocking Blue
1971	Nino Bravo - Te quiero	George Harrison - My sweet lord
1972	Mari Trini - Yo no soy esa	Redbone - The witch queen of New Orleans
1973	Mocedades – Eres tú	Wings - Hi, hi, hi
1974	Juan Pardo - Conversaciones Conmigo Mismo	Suzi quatro - 48 crash
1975	Camilo Sesto - Melina	Barry White - You're my first my last my everything
1976	Santa Barbara - Donde estan tus ojos negros	Silver Convention - Fly Robin fly
1977	Raffaella Carrá_Fiesta	Boney M – Ma Baker
1978	Daniel Magán – Cara de gitana	Bee Gees - Night fever
1979	Víctor Manuel – Sólo pienso en tí	Patrick Hernandez - Born to Be Alive
1980	Los Pecos - Háblame de ti	The Buggles, Video killed The Radio star
1981	Maria Jesus - El baile de los pajaritos	Robert Palmer - Johnny and Mary
1982	Mecano - Me cole en una fiesta	Lime - Babe we're gonna love tonight
1983	Azul y Negro – No tengo tiempo	The Police - Every breath you take
1984	La unión - Hombre lobo en Paris	Michael Jackson - Thriller
1985	Miguel Bose - Amante Bandido	Opus - Live is life
1986	Gabinete Caligari - El calor del amor en un bar	Lionel Richie - Say you, Say me
1987	DunCan Dhu - Jardin de Rosas	The Bangles - Walk Like An Egyptian
1988	Tino Casal - Eloise	U2 - Desire
1989	The Refrescos – Aquí no hay playa	Kaoma - Lambada
1990	Radio Futura - Veneno en la piel	MC Hammer - U can't touch this
1991	Héroes del Silencio - Entre dos tierras	Bryan adams - Everything I do
